# Using deep learning to predict survival outcome in non-surgical cervical cancer patients based on pathological images

**DOI:** 10.1007/s00432-022-04446-8

**Published:** 2023-01-19

**Authors:** Kun Zhang, Kui Sun, Caiyi Zhang, Kang Ren, Chao Li, Lin Shen, Di Jing

**Affiliations:** 1 Department of Oncology, National Clinical Research Center for Geriatric Disorders, Xiangya Hospital, Central South University, Changsha, 410008 China; 2 Department of Radiology, Shandong Provincial Hospital Affiliated to Shandong First Medical University, Jing Wu Road, No. 324, Jinan, 250021 China; 3 Department of Obstetrics and Gynecology, Maternal and Child Health Hospital of Hunan Province, Changsha, 410008 China; 4 Department of Radiation Oncology, Peking Union Medical College Hospital, Chinese Academy of Medical Sciences & Peking Union Medical College, Beijing, 100730 China; 5 Department of Radiation Oncology, Shenzhen People’s Hospital, The First Affiliated Hospital of Southern University of Science and Technology, Shenzhen, 518020 Guangdong China

**Keywords:** Cervical cancer, Radiochemotherapy, Deep learning, Overall survival, Pathological images

## Abstract

**Purpose:**

We analyzed clinical features and the representative HE-stained pathologic images to predict 5-year overall survival via the deep-learning approach in cervical cancer patients in order to assist oncologists in designing the optimal treatment strategies.

**Methods:**

The research retrospectively collected 238 non-surgical cervical cancer patients treated with radiochemotherapy from 2014 to 2017. These patients were randomly divided into the training set (*n* = 165) and test set (*n* = 73). Then, we extract deep features after segmenting the HE-stained image into patches of size 224 × 224. A Lasso–Cox model was constructed with clinical data to predict 5-year OS. C-index evaluated this model performance with 95% CI, calibration curve, and ROC.

**Results:**

Based on multivariate analysis, 2 of 11 clinical characteristics (C-index 0.68) and 2 of 2048 pathomic features (C-index 0.74) and clinical–pathomic model (C-index 0.83) of nomograms predict 5-year survival in the training set, respectively. In test set, compared with the pathomic and clinical characteristics used alone, the clinical–pathomic model had an AUC of 0.750 (95% CI 0.540–0.959), the clinical predictor model had an AUC of 0.729 (95% CI 0.551–0.909), and the pathomic model AUC was 0.703 (95% CI 0.487–0.919). Based on appropriate nomogram scores, we divided patients into high-risk and low-risk groups, and Kaplan–Meier survival probability curves for both groups showed statistical differences.

**Conclusion:**

We built a clinical–pathomic model to predict 5-year OS in non-surgical cervical cancer patients, which may be a promising method to improve the precision of personalized therapy.

## Introduction

Cervical cancer is the most common gynecologic malignancy (Sung 2021; Ferrall et al. 2021). In 2018, it was estimated that 570,000 women were diagnosed with cervical cancer worldwide and about 311,000 cancer-related mortality resulted from the disease (Arbyn et al. 2020). According to NIH, the 5-year overall survival (OS) rate of all the cases between 2012 and 2018 in the United States is still below 66.7%, and improving the OS remains a challenge. Currently, the standard first-line cervical cancer treatments include surgery, surgery followed by chemotherapy or/and radiotherapy (Koh et al. 2019). How to select the aforementioned treatments mainly depended on the pathological classification, T stage and N stage of the disease. To this end, precise prediction of clinical outcomes is necessary for individualized treatment, which could provide the optimal scheme. The goal of the algorithm we developed is to better distinguish the different prognoses in cervical cancer patients (Matsuo et al. 2019).

Pathologic biopsy is recognized as the golden standard for measuring malignant neoplasms and HE staining is the most used method in the routine (Coudray et al. 2018). Even though an elaborate classification system for cervical cancer has already been developed, the prognosis evaluation is still clouded by disagreements in terms of interpretation and judgments among pathologists (Ke et al. 2021; Yuan Yuan et al. 2020). Generally, traditional hazard models have been utilized to evaluate survival. Although it is feasible to predict patients’ OS, these models have analyzed the difference of patients in cohorts and not survival outcomes. Deep learning (DL) models have developed rapidly in the processing of medical images in recent years. These models have been widely utilized in the classification of pathological diagnosis (Yamashita et al. 2021; Bera et al. 2019; Zhang et al. 2022) and prediction of treatment response, but the survival prediction remains limited. Establishing an evidence-based, objective model to predict survival outcomes is crucial.

Whole slide imaging (WSI), as an advanced digital pathology, has recently received much attention for its utility in cancer diagnosis. WSI combined with convolutional neural networks (CNN) algorithms can significantly simplify the process of pathological diagnosis and alleviate the burden on pathologists. It has already been utilized in measuring multiple cancers. Based on WSI and clinical data, an interpretable, weakly supervised deep-learning framework has been proposed by Shi et al. (2021), providing a valuable meaning for the stratification of HCC risk and prediction of treatment response. Coudray et al. (2018) also showed that CNN applied to diagnose the major histological subtypes of non-small cell lung cancer (NSCLC) and predict the mutational status of various genes (e.g., serine/threonine kinase 11, epidermal growth factor receptor) based on the pathological WSI. However, there are still no established standards for its practice in the field due to the limitations of the storage space and scanning equipment. In addition, the images are often oversized leading to difficulties in transfer between devices and networks (Wilbur 2011; Noorbakhsh et al. 2020). The most representative HE-staining images extracted from WSIs could contain similar cytological characteristics carrying hide features information to access and analyze (Yu et al. 2016). Currently, there are already reports of utilizing H&E staining combined with molecular markers and deep-learning models to differentiate glioblastomas (Kather et al. 2019; Jin et al. 2021). So far as we know, using the HE diagnostic images to predict the survival outcomes of cervical cancer patients has not been reported.

**Fig. 1:**
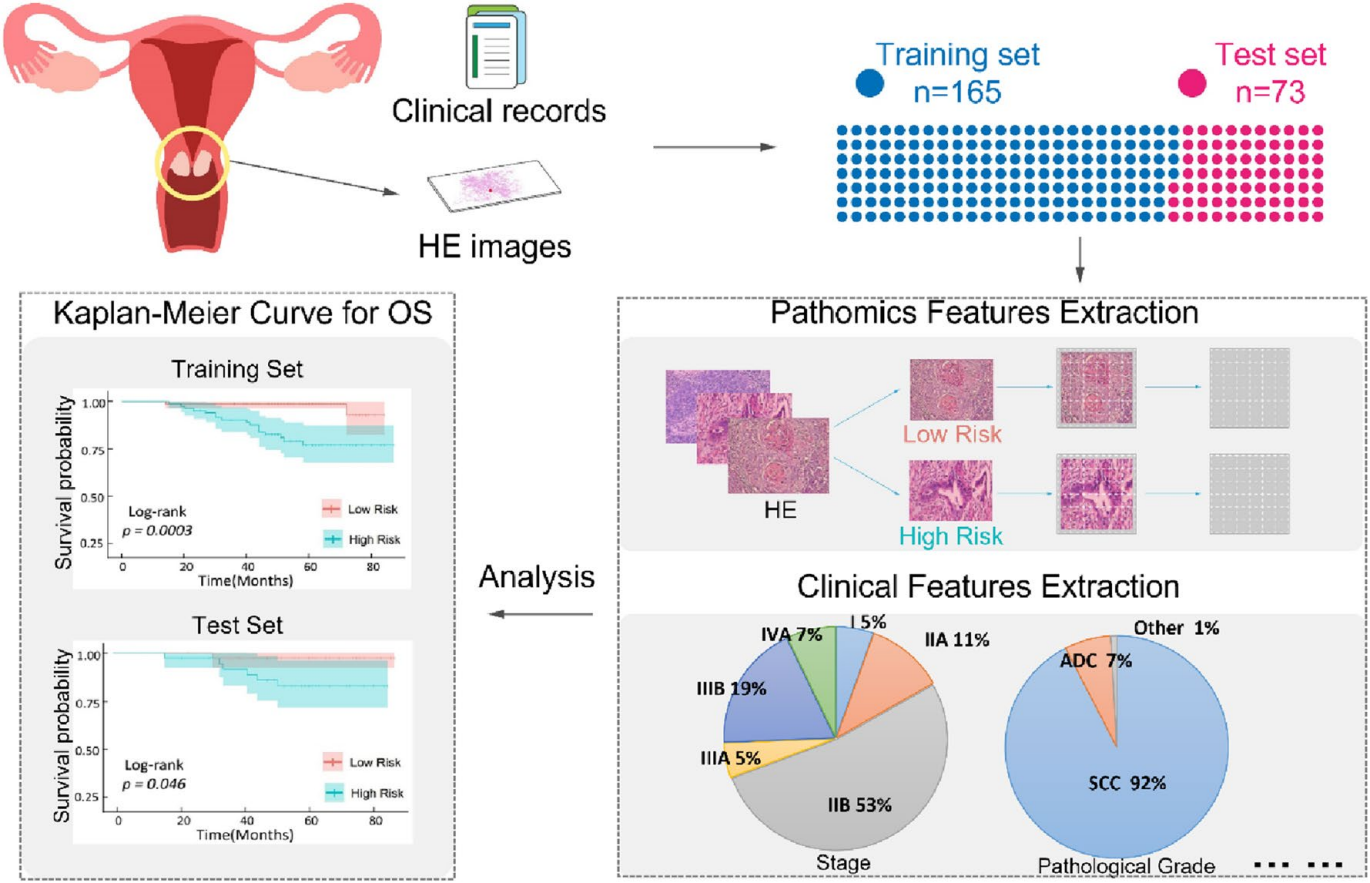
The schematic of collection and analyzation of cervical cancer pathological images and clinicopathologic characteristics based on deep learning

In this study, we chose the classic HE-staining images in combination with clinicopathologic characteristics to establish a deep-learning model (Fig. 1) for the prediction of clinical outcomes to assist oncologists in designing therapeutic regimens and optimizing follow-up time.

## Methods

### Datasets

A total of 238 patients diagnosed with cervical cancer were recruited from March 2014 to December 2017 in Xiangya hospital. Ethical approval was obtained by the Ethics Committee of Xiangya Hospital (No.202206143) and waived informed consent was granted. These non-surgical cervical cancer patients staged IB2-IVA and were treated with external beam radiotherapy (EBRT) plus brachytherapy in combination with the current chemotherapy. The diagnostic pathological images were obtained from an experienced pathologist.

### Patch cutting and color standardization and deep feature extraction

The pathological images of cervical cancer were cut into patches of size 224 × 224. Considering the problem that uneven staining of slices would cause errors in the model, the Vahadane et al. (2016) method was used to standardize the color of pathological patches. After the patch color standardization process was completed, using ResNet-152 (Hwang et al. 2016) with pre-trained weights in ImageNet (https://www.image-net.org/) as the backbone network, deep feature extraction was performed on each color-normalized patch. After the computation of convolutional layers, hidden layers and fully connected layers, 2048 deep features of each patch were extracted. Considering that a patient with multiple patches would cause a certain degree of confusion, we calculated the median of deep features of multiple patches per person was used as the deep feature value representing the individual. The deep network framework was built on Python version (3.8.0) with Pytorch version (1.11.0).

### Dataset partitioning and feature screening

A total of 238 patients were randomly assigned to the 2 datasets with a ratio of 7:3, with 165 people in the training set and 73 people in the test set. The Least Absolute Shrinkage and Selection Operator (LASSO) algorithm was invented by Robert Tibshirani in 1996 and it had already become a standard method for feature selection in the field of bio-information (Vidyasagar 2015). It had the merit of utilizing L1 regularization to reduce the possibility of redundancy and overfitting in regression. Thus, we chose Lasso–Cox as our regression model for feature selection.

**Fig. 2:**
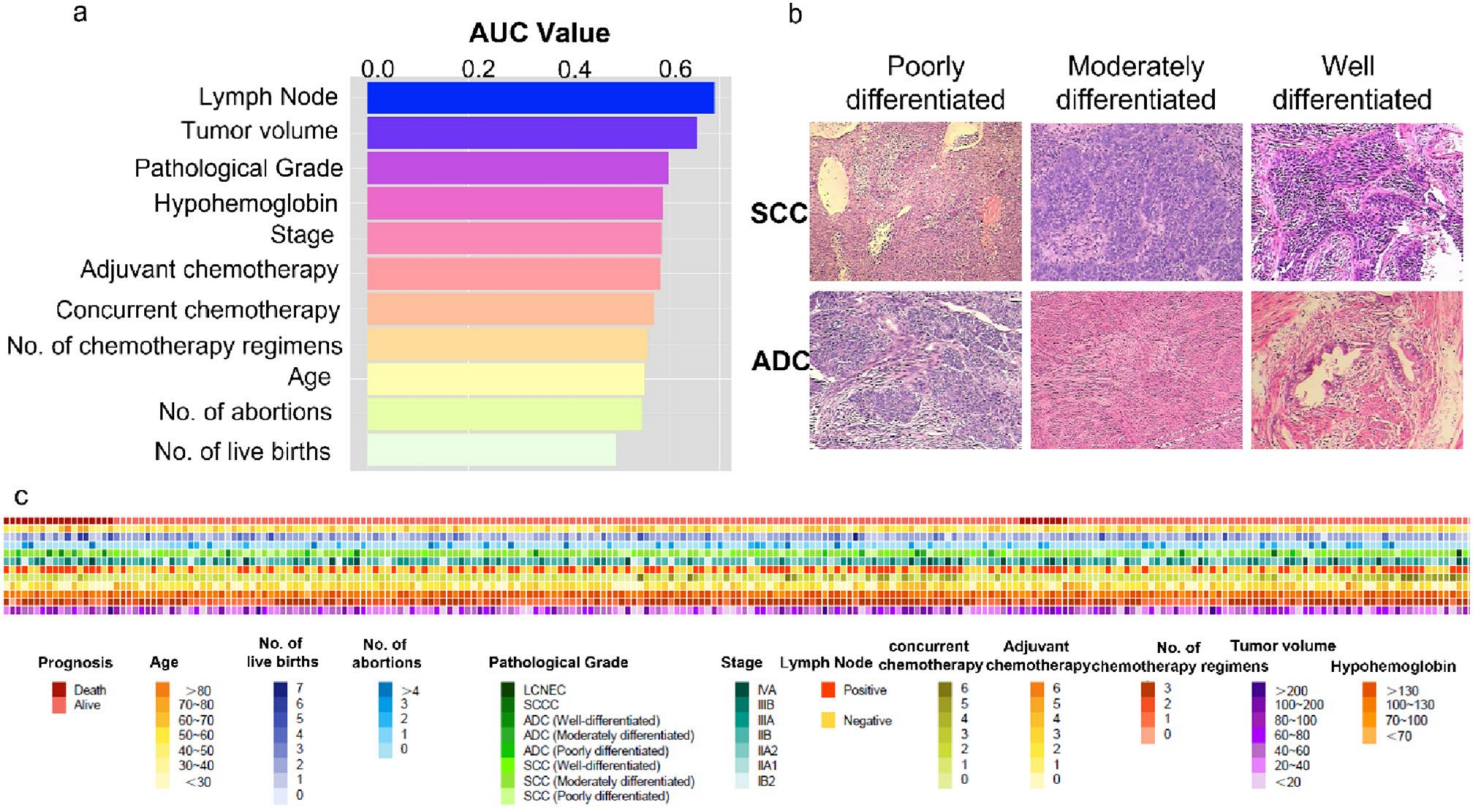
Extraction of clinical and pathological characteristics. **a** The color of pathological slice patches were standardized and 11 clinical features (AUROC > 0.6) were screened to construct a Lasso–Cox model via ResNet-152. **b** Pathological grades of cervical cancer, *SCC* squamous cell carcinoma, *ADC* adenocarcinoma. **c** Heat map of 238 cervical cancer patients with clinical characteristics

First, we standardized 2048 deep features using Z-score to ease the convergence process. Then, we fed the Lasso–Cox model with the data in the training set and randomly assigned 100 penalty parameters lambda for the training. Last, we utilized the tenfold cross-validation method to determine the standard error of lambda and used it as the optimal penalty parameter to select the optimal deep features. The evaluation index was C-index. In the process of selecting clinical features, we chose each feature individually to establish a Cox model and used AUROC (the area under the receiver operator characteristic curve) for evaluation. The features with a score above 0.6 were viewed as valuable (Fig. 2A). The randomization process of datasets and Lasso–Cox model establishment was conducted with the “caret” and “glmnet” package of the R software (version 4.1.2).

### Cox model development and evaluation

We had established three Cox models, including the optimal pathomic features, clinical characteristics, and the combination of the two elements, to predict the patients with 5-year OS. The models were evaluated by C-index, calibration curve, and ROC curve. The visualization of Cox models was conducted with Nomogram. The calibration curve, ROC curve, and Nomogram were conducted with the “rms” and “pROC” package of the R software (version 4.1.2).

## Result

### Clinical characteristics

This research retrospectively collected 238 locally advanced cervical cancer patients treated with radiochemotherapy from 2014 to 2017 in Xiangya Hospital. The diagnostic pathological images and clinical characteristics were collected for analysis. The clinical characteristics of these cases are shown in Table 1. Those data were divided into a training set of 165 cases and a test set of 73 cases.

**Table 1:** Characteristics of non-surgical cervical cancer patients

Characteristics	Training set (*n* = 165)	Testing set (*n* = 73)
Age		
Range	21–83	34–67
Median	51	52
Histological type		
SCC	150 (91%)	69 (94.4%)
ADC	14 (8.4%)	2 (2.8%)
Others	1 (0.6%)	2 (2.8%)
FIGO stage		
I	12 (7.3%)	2 (2.7%)
II	112 (67.9%)	45 (61.6%)
III	30 (18.2%)	19 (26.1%)
IV	11 (6.6%)	7 (9.6%)
Tumor volume (cm^3^)		
< 20	21 (12.7%)	7 (9.6%)
20–60	89 (54%)	33 (45.3%)
60–100	35 (21.2%)	24 (32.8%)
≥ 100	20 (12.1%)	9 (12.3%)
Lymph node		
Positive	90 (54.5%)	37 (50.7%)
Negative	75 (45.5%)	36 (49.3%)
No. of live births		
None	2 (1.2%)	1 (1.4%)
1–3	147 (89.2%)	67 (91.8%)
≥ 4	16 (9.6%)	5 (6.8%)
No. of abortion		
None	117 (70.9%)	54 (74.0%)
1–3	36 (21.8%)	16 (21.9%)
≥ 4	12 (7.3%)	3 (4.1%)
Concurrent chemotherapy		
None	19 (11.5%)	3 (4.1%)
Yes	146 (88.5%)	70 (95.9%)
Adjuvant chemotherapy		
None	43 (26.1%)	18 (24.7%)
Yes	122 (73.9%)	55 (75.3%)
Hypohemoglobin		
< 70	1 (0.6%)	0
70–100	11 (6.6%)	9 (12.3%)
100–130	77 (46.7%)	36 (49.3%)
≥ 130	76 (46.1%)	28 (38.4%)

*FIGO* International Federation of Gynecology and Obstetrics, *SCC* Squamous carcinoma, *ADC* Adenocarcinoma; histological type—others include 1 small cell carcinoma (SCCC) and 2 large cell neuroendocrine carcinoma

### Optimal features’ generation for model

According to the clinical characteristics we collected, Cox models were established, respectively, and these evaluation indexes included age, number of live births, abortion, pathological grade, clinical stage, and lymph node metastasis. Two clinical characteristics (lymph nodes metastasis and tumor volume) were screened out by AUROC > 0.600 as the evaluation index (Fig. 2a). In the deep features’ selection, two features we named No.769 and No.1409 were preferred out of the 2048 deep features. This part was done using the Lasso–Cox algorithm with the best penalty coefficients. In Fig. 2b, we listed the pathological grades of cervical cancer. The clinical characteristics of the patients are shown in Fig. 2c.

### Credibility analysis and validation of the combined model

**Fig. 3:**
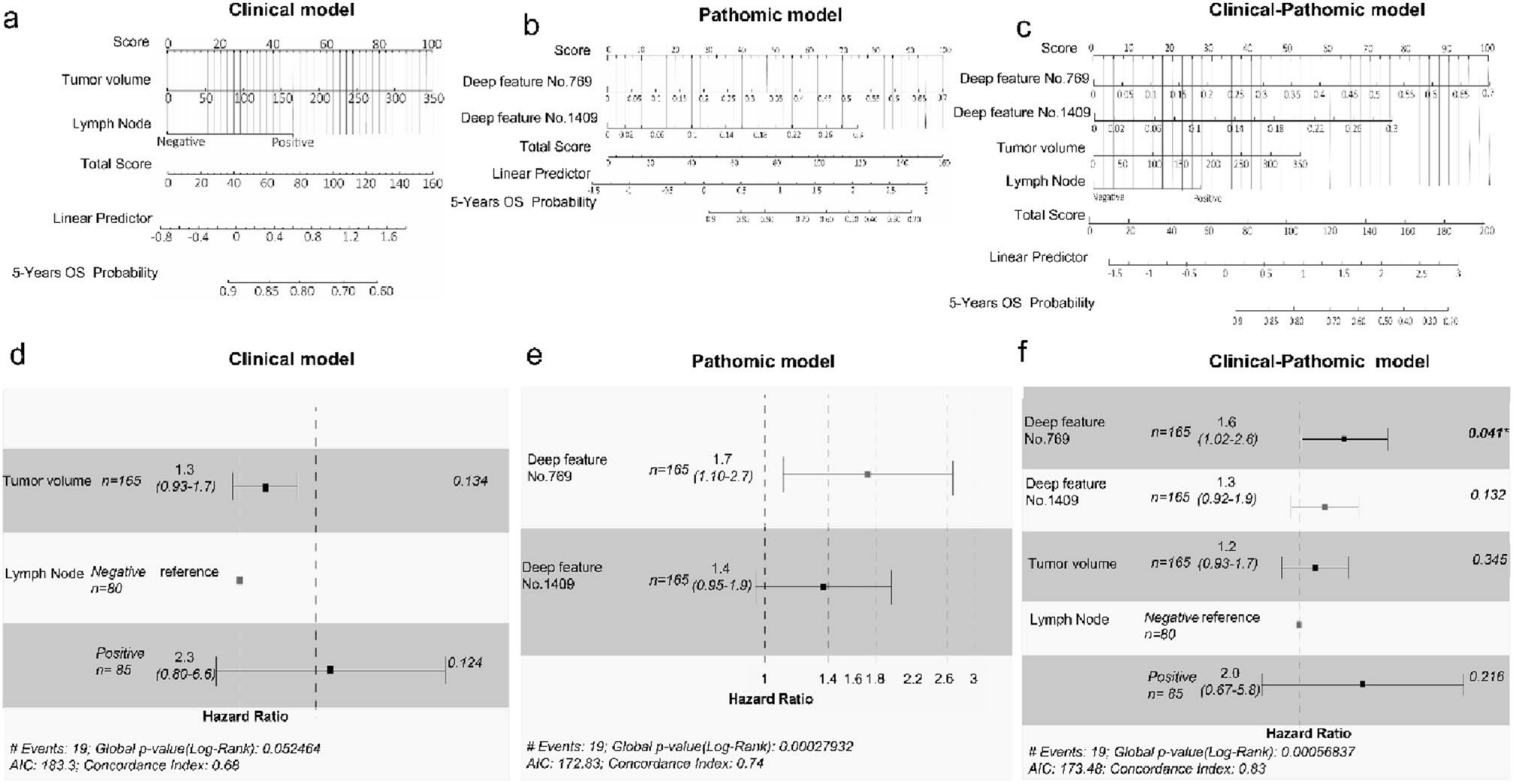
Credibility analysis of the 5-year survival prediction models. **a–c** The nomograms for predicting the 5-year survival in cervical cancer based on clinical model, the pathomic model, the clinical–pathomic model. **d–f** The forest plots of characteristic risk scores to analyze survival outcome in clinical model, the pathomic model and the clinical–pathomic model

**Fig. 4:**
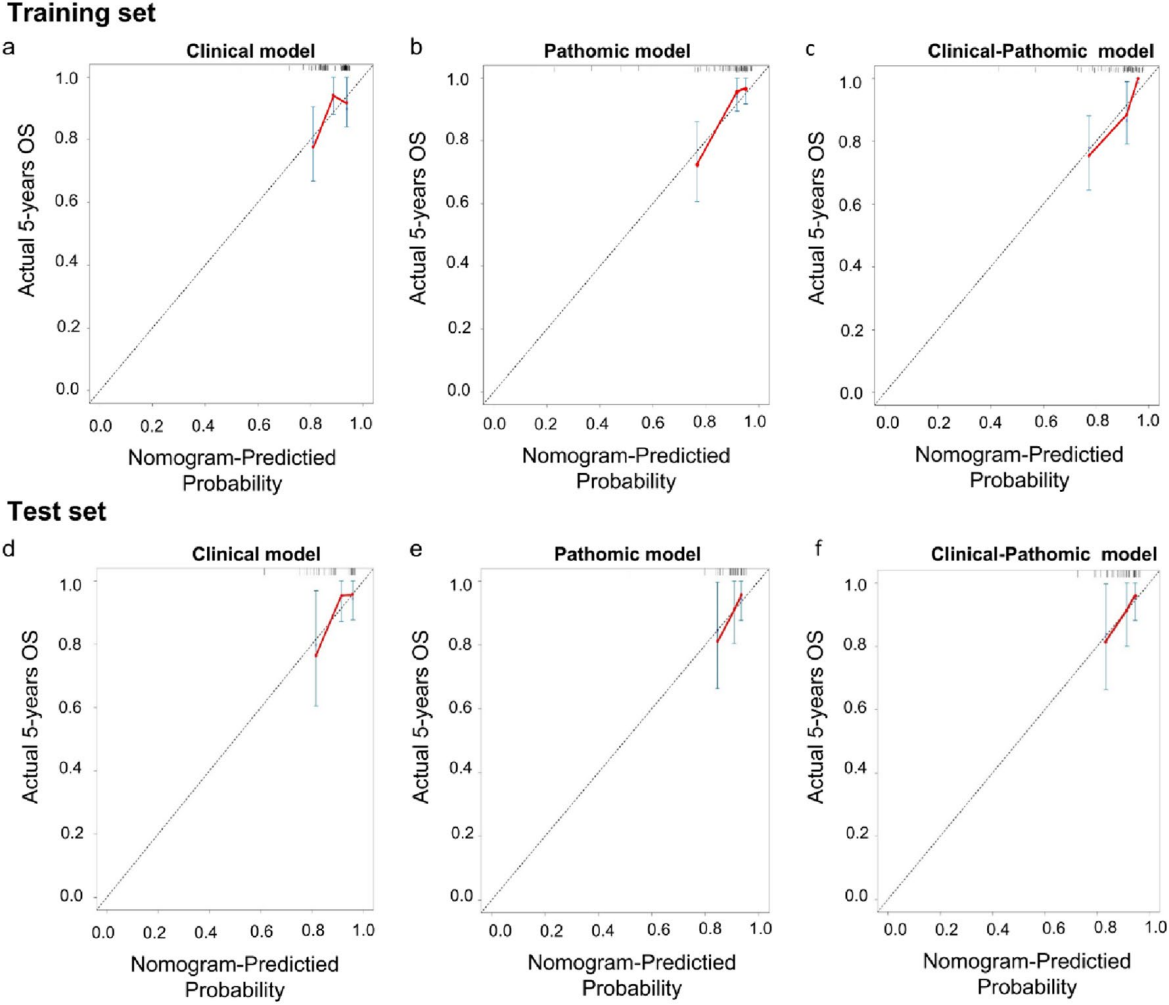
Calibration curves of the clinical model, the pathomic model, and the clinical–pathomic model in the training set **(a–c)** and in the test set **(d–f)**

We separately constructed three models to predict 5-year OS (Fig. 3a–c). The clinical model integrated two optimal clinical characteristics, and the pathomic model was composed of two valuable deep features. Moreover, we combined the above clinical and deep features to discover whether the clinical–pathomic model was superior to others. The clinical model showed good performance to predict 5-year OS for non-surgical cervical cancer patients, with C-indexes of 0.680 (95% CI 0.543–0.811) (Fig. 3d) in the training set and 0.710 (95% CI 0.556–0.863) in the test set. The pathomic model showed efficacy near to clinical model, with C-indexes of 0.746 (95% CI 0.608–0.883) (Fig. 3e) in the training set and 0.700 (95% CI 0.514–0.887) in the test set. The performance of the clinical–pathomic model was better than the above single-model both in the training and test set, with C-indexes of 0.835 (95% CI 0.759–0.912) and 0.737 (95% CI 0.555–0.918) (Fig. 3f). The calibration curve of the nomograms is shown in Fig. 4a–c, which showed good agreement between the prediction of 5-year survival outcome in the training set and in the test set (Fig. 4d–f).

### Performance of the combined model

**Fig. 5:**
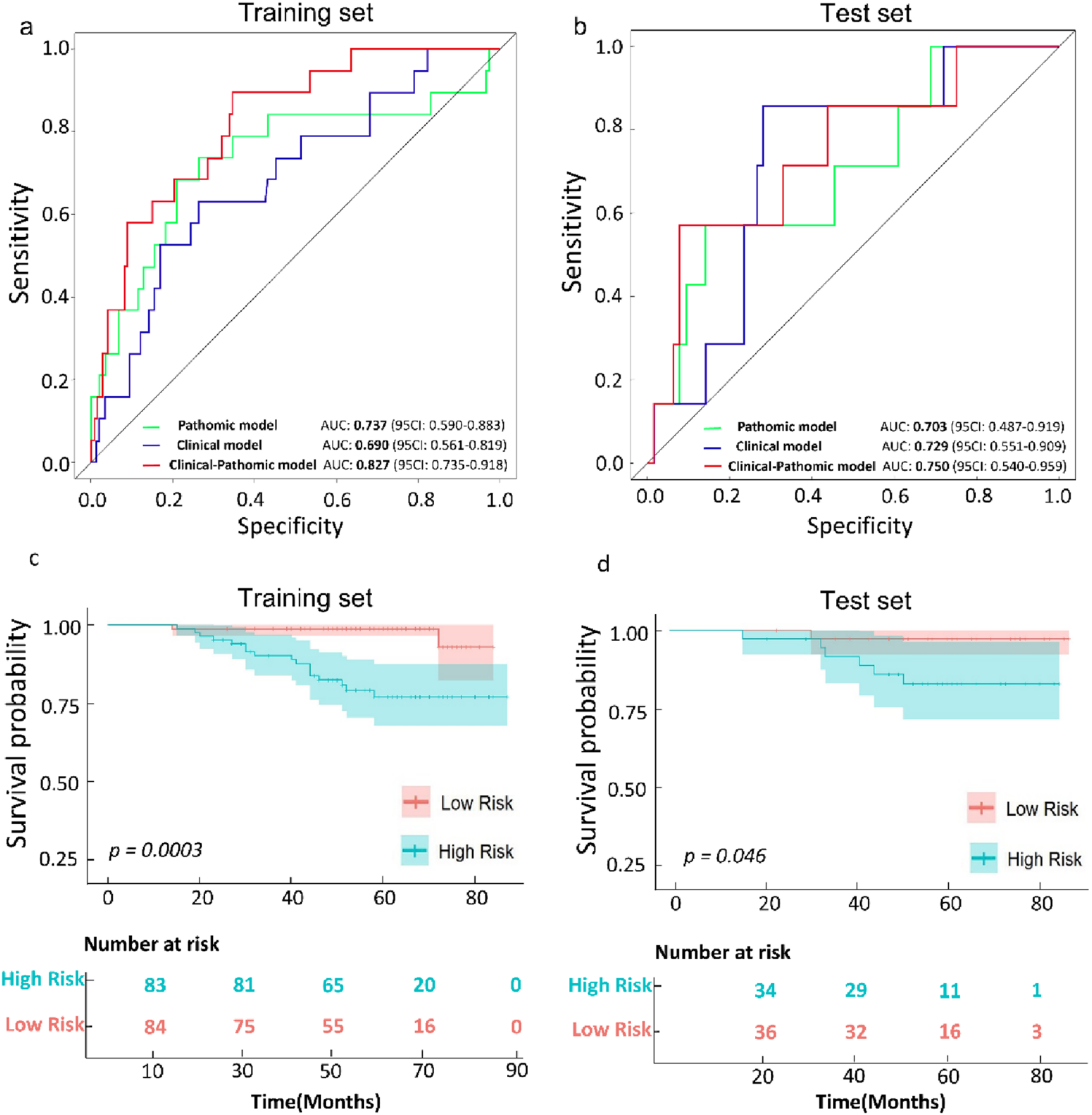
Performance and validation of the 5-years survival prediction models. **a–b** The accuracy of three models for predicting the 5-year survival rate of cervical cancer detected by ROC curve in the training set and the test set. **c–d** Kaplan–Meier curves of 5-year survival between low-risk and high-risk groups defined by nomogram of the clinical–pathomic model in the training set and the test set

In comparison to the features of pathological images and clinical characteristics used alone, the clinical–pathomic model demonstrated a larger area under the 5-year survival prediction curve (Fig. 5a–b). As shown in Fig. 5a, the developed nomogram exerted a powerful predictive ability in the training set with AUCs of 0.827 (95% CI 0.735–0.918), respectively, which were higher than the pathomic model (AUC = 0.737, 95% CI 0.590–0.883) and clinical model (AUC = 0.690, 95% CI 0.561–0.819). We performed validation on the test set and found the same result. The clinical–pathomic model’s AUC was 0.750 (95% CI 0.540–0.959), while the AUC of the clinical model was 0.729 (95% CI 0.551–0.909), and the AUC of the pathomic model was 0.703 (95% CI 0.487–0.919). Figure 5c–d depicts the performance of each risk score. To utilize the combined model, we divided the patients into a high-risk and low-risk groups based on the appropriate nomogram scores. In every set, the Kaplan–Meier survival probability curves for the two groups showed statistical divergence (*p* = 0.0003 for training set, *p* = 0.046 for test set). The combined model showed the predictive power for 5-year OS.

## Discussion

In this retrospective study, we demonstrated that the utilization of pathological diagnostic images to predict 5-year survival outcomes via a deep-learning approach performed superior ability in non-surgical cervical cancer patients. Most of this research mainly focused on either the analyzing radiographic images for predicting treatment response or detecting abnormal cells from tissue slides for classification and biomarkers discovery. The study of predicting 5 years of survival in non-surgical cervical cancer by deep-learning model remains limited. In the field of cervical cancer, the application of the deep-learning model has been studied for detecting human papillomavirus (HPV) (Kahng et al. 2015), examination of cytologic testing (Komagata et al. 2017), screening cervical carcinoma via colposcopy pictures (Hu et al. 2019), and prediction of radiochemotherapy response, but only several studies related to overall survival.

Although surgery is the cornerstone in early-stage cervical cancer, the prognostic of postoperation has been significantly affected by surgical techniques, equipment, nutritional level, and nursing competence. Dr. Kluska and his colleagues found that utilization of deep-learning model predicted 5 years of survival outcomes based on clinicopathologic characteristics from cervical cancer patients treated with radical hysterectomy. Nevertheless, with the development of radiochemotherapy, radical surgery and radiochemotherapy achieved similar prognostic in early-stage cervical cancer patients (Landoni et al. 2017). Advanced-stage cervical cancer included radiochemotherapy as the standard therapeutic regimen (Cohen et al. 2019). Utilization of the deep-learning model based on the pathological images and prediction of the 5-year survival rate in non-surgical cancer patients were more meaningful results and predictability. The pathological images were segmented into patches. Then, normalization and extraction features from the patches were used to construct the Lasso–Cox models.

In recent years, deep learning has been used in various fields, such as predicting chemotherapy response, lymph node metastasis via radiomics (Peng et al. 2019; Li et al. 2020; Lao 2017) and analyzing pathological images. Many studies have shown promising results for classification and prediction, including prediction of gene mutation in the adenocarcinoma of non-small cell lung cancer (Coudray et al. 2018), development of a risk-strategies model in hepatocellular carcinoma (HCC)(Saillard et al. 2020), classification of gastric epithelial tumors (Park et al. 2021), differentiation of breast cancer subtypes (Han et al. 2017), and detection of brain vasculature (Todorov et al. 2020). Although there have been reported several radiomics studies in cervical cancer, histopathology of the deep-learning model only focuses on screening of cytologic testing, which is the leading cause of cancer-related death among women worldwide (Jin et al. 2021; Sung 2021). Therefore, it is essential to develop high-performance algorithms with high sensitivity and specificity to predict the prognosis of cervical cancer. The deep-learning model of pathological images for predicting overall survival reflected the heterogeneity of tumor sites. In addition, the cytological features could contain much-hidden information closely related to OS, which plays the most important role in the evaluation of treatment response. Nevertheless, few relevant deep-learning models have been found in cervical cancer. By studying a variety of deep-learning strategies, the model we developed gave relatively reliable results with relatively high accuracy.

Although the results of our 5-year predictive models are very promising for translation into clinical practice, there is still room for improvement in the future. (i) This retrospective study has a relatively small sample size from a single institution. A large amount of sample size should be included from multi-centers to design randomized clinical trials. (ii) The standardization of sample preparation is critical and closely related to the accuracy of the model, such as tissue sectioning, staining, acquisition of representative tumor areas, and the quality of the pathological image. How to distinguish different cellular components (tumor cells, lymphocytes, red blood cells, etc.) in the same patch should be further investigated. (iii) More specific biomarkers and clinical parameters (diabetes, hypertension, and other diseases) should be integrated into the survival prediction model to improve the accuracy.

The deep-learning model based on representative pathologic features and clinical data had a superior performance for the predicting 5-year OS in cervical cancer and showed great clinical application promising.

## Conclusion

In conclusion, we developed a deep-learning model based on pathological features and clinical risk factors, which can effectively predict the 5-year survival outcome in non-surgical cervical cancer patients treated with radiochemotherapy.

## Data Availability

The datasets used to support this finding of study are included in the article.
